# Machine learning-based prediction of PASI100 response to secukinumab in patients with psoriasis: a real-world study with SHAP interpretability analysis

**DOI:** 10.3389/fmed.2026.1764025

**Published:** 2026-02-27

**Authors:** Fengming Hu, Jian Gong, Yuxin Li, Xiaohua Tao, Lihua Zhang

**Affiliations:** 1 Dermatology Hospital of Jiangxi Province, Nanchang, China; 2 The Affiliated Dermatology Hospital of Nanchang University, Nanchang, China; 3 School of Public Health and Health Management, Gannan Medical University, Ganzhou, China

**Keywords:** machine learning, PASI100, psoriasis, random forest, real-world evidence, secukinumab, SHAP, treatment response prediction

## Abstract

**Background:**

Secukinumab, an interleukin-17A (IL-17A) inhibitor, has demonstrated significant efficacy in treating moderate-to-severe plaque psoriasis. Achieving complete skin clearance (PASI 100) is the ideal therapeutic goal. However, individual responses vary, and tools to accurately predict PASI 100 response in real-world settings are lacking.

**Methods:**

In this retrospective study, we analyzed data from 11,134 psoriasis patients who were treated with secukinumab for 3 months. The dataset was randomly split into training (70%) and testing (30%) sets. Univariate analysis and LASSO regression were used for feature selection. Eight machine learning algorithms, including Random Forest, LightGBM, and Logistic Regression, were developed to predict treatment response. Model performance was evaluated using the Area Under the Receiver Operating Characteristic Curve (AUC). SHapley Additive exPlanations (SHAP) analysis was employed to interpret the optimal model.

**Results:**

A total of 4,593 (41.25%) patients achieved PASI 100 response. The factors of Disease duration, BMI, bBSA, bPASI, bDLQI, Gender, bIGA, Education background, Job status, Comorbidity, Family history, Drug allergy history, Disease situation, Traditional systemic therapy, Medical insurance, Disease status and Biologic usage status were significantly associated with PASI 100 response (all *p* < 0.05), while others not. LASSO regression identified 5 key predictors, including Gender, bIGA, bBSA, bPASI and bDLQI. Among the algorithms, Random Forest (training AUC = 0.879, testing AUC = 0.757) and LightGBM (training AUC = 0.834, testing AUC = 0.761) demonstrated the best performance in those machine learning algorithms. SHAP analysis revealed that gender and baseline disease severity indicators (bIGA, bBSA, bPASI and bDLQI) were important predictors.

**Conclusion:**

We successfully developed Random Forest and LightGBM-based prediction model for PASI100 response to secukinumab with moderate discriminative ability. Baseline disease severity emerged as the dominant predictor of complete skin clearance. These findings provide evidence-based support for personalized treatment goal setting and patient selection in clinical practice.

## Introduction

Psoriasis is a chronic, immune-mediated inflammatory skin disease affecting approximately 2–3% of the global population, characterized by erythematous, scaly plaques that significantly impair patients’ quality of life (1, 2). The pathogenesis of psoriasis involves complex interactions between genetic susceptibility, environmental triggers, and immune dysregulation, with the interleukin-23/interleukin-17 (IL-23/IL-17) axis playing a central role in disease development and maintenance (3). Secukinumab, a fully human monoclonal antibody that selectively neutralizes IL-17A, represents a major advancement in psoriasis treatment. Pivotal clinical trials including ERASURE and FIXTURE demonstrated that secukinumab achieved PASI75 response rates exceeding 80% and PASI90 response rates of approximately 60% at week 12 (4). Subsequent studies have established secukinumab as one of the most effective biologics for achieving complete skin clearance, with PASI100 response rates ranging from 30 to 45% depending on patient populations and follow-up duration (5, 6).

Complete skin clearance (PASI100) has emerged as the optimal treatment target in contemporary psoriasis management. Patients achieving PASI100 report significantly greater improvements in quality of life, psychological wellbeing, and treatment satisfaction compared to those achieving PASI75 or PASI90 (7). The concept of “treat-to-target” has been increasingly advocated, with PASI100 representing the ultimate therapeutic goal (8). However, despite the overall high efficacy of secukinumab, considerable inter-individual variability exists in treatment response, and not all patients achieve complete clearance.

Identifying factors associated with PASI100 response is clinically important for several reasons. First, it enables personalized treatment goal setting based on individual patient characteristics. Second, it facilitates informed shared decision-making between clinicians and patients regarding treatment expectations. Third, it may help optimize healthcare resource allocation by identifying patients most likely to benefit from specific therapies (9). Previous studies have identified several factors potentially associated with biologic response, including body weight, disease duration, previous biologic exposure, and baseline disease severity (10–12). However, these studies have generally employed traditional statistical methods that may not capture complex, non-linear relationships between predictors and outcomes.

Machine learning algorithms offer advantages over conventional statistical approaches in handling high-dimensional data, capturing non-linear relationships, and modeling complex interactions between variables (13). These methods have been increasingly applied in dermatology for diagnostic and prognostic purposes, including skin cancer detection, atopic dermatitis severity assessment, and psoriasis diagnosis (14, 15). However, their application in predicting biologic treatment response in psoriasis remains limited.

A critical challenge in applying machine learning to clinical decision-making is the “black box” nature of many algorithms, which limits interpretability and clinical acceptance (16). SHapley Additive exPlanations (SHAP), based on cooperative game theory, provides a unified approach to interpreting predictions by quantifying each feature’s contribution to individual predictions (17). This method has been increasingly adopted in medical machine learning studies to enhance model transparency and clinical utility.

The primary objective of this study was to develop and compare multiple machine learning models for predicting PASI100 response to secukinumab using real-world data from a large cohort of psoriasis patients. Secondary objectives included identifying core predictive factors through feature selection and elucidating the mechanisms underlying prediction through SHAP interpretability analysis.

## Materials and methods

### Study design and data source

This was a retrospective cohort study utilizing real-world clinical data from a multicenter database. The patients with psoriasis who received secukinumab treatment at the Chinese Psoriasis Standardized Diagnosis and Treatment Center database from June 2020 to September 2024, and all patients sign informed consent forms upon entry into the database. Inclusion criteria were: (1) diagnosis of plaque psoriasis; (2) treatment with secukinumab; and (3) availability of baseline and follow-up PASI scores. The primary outcome was achieving PASI 100 (100% improvement from baseline PASI) after a defined treatment period. Baseline characteristics included demographic data (age, gender, BMI, education, job, marriage, region), disease characteristics (duration, disease status, family history, comorbidities, history of malignancy, allergy history), and clinical assessments (bPASI, bBSA, bDLQI, bIGA). Treatment history (topical therapy, biologic usage status, traditional systemic medications) and medical insurance status were also recorded.

### Study population

Patients were eligible for inclusion if they met the following criteria: (1) age ≥18 years; (2) clinical and/or histopathological diagnosis of plaque psoriasis; (3) received standard-dose secukinumab treatment (300 mg subcutaneously at weeks 0, 1, 2, 3, and 4, followed by more than 3 months maintenance); and (4) had complete baseline and follow-up data available. Exclusion criteria included: (1) concurrent diagnosis of other psoriasis subtypes (pustular, erythrodermic, or guttate psoriasis); (2) pregnancy or lactation; (3) severe systemic comorbidities that could interfere with efficacy assessment; and (4) loss to follow-up or substantial missing data.

### Outcome definition

The primary outcome was PASI100 response, defined as achievement of 100% improvement from baseline PASI score (complete skin clearance) at the designated assessment time point.

### Predictor variables

Candidate predictor variables were selected based on clinical relevance and data availability, encompassing five domains: (1) Demographic characteristics: age, sex, body mass index (BMI), education level, occupation, marital status, and insurance status. (2) Disease-related characteristics: disease duration, baseline body surface area involvement (bBSA), baseline PASI (bPASI), baseline Investigator Global Assessment (bIGA), baseline Dermatology Life Quality Index (bDLQI), disease status (new-onset vs. recurrent), family history of psoriasis, presence of comorbidities, and history of malignancy. (3) Allergy history: history of drug allergy and history of allergic diseases. (4) Treatment history: prior topical therapy, prior conventional systemic therapy (including methotrexate, cyclosporine, and acitretin) and biologic usage status (biologic-experienced vs. biologic-naïve). (5) Lifestyle factors: smoking status. (6) Geographic factors: China region (including North area and South area).

### Statistical analysis and feature selection

Continuous variables were compared between PASI100 response and non-response using independent samples *t*-test. Categorical variables were compared using Pearson’s chi-square test. Variables with *p* < 0.01 in univariate analysis were considered for inclusion in the feature selection process. To reduce overfitting risk and identify the most informative predictors, LASSO regression with 10-fold cross-validation was performed. The optimal regularization parameter (*λ*) was determined based on the λ.1se criterion (the largest λ within one standard error of the minimum cross-validation error). The relationship between the number of features and model AUC was plotted to guide final feature selection, balancing predictive performance, clinical interpretability, and practical applicability.

### Machine learning model development and evaluation

The dataset was randomly partitioned into training (70%) and testing (30%) sets, stratified by outcome. Continuous variables were standardized using z-score normalization. Categorical variables were encoded using one-hot encoding. Eight ML algorithms were implemented: Decision Tree (DT), Random Forest (RF), Multi-layer Perceptron (MLP), Radial Basis Function Support Vector Machine (RBF-SVM), eXtreme Gradient Boosting (XGBoost), Logistic Regression (LR), K-Nearest Neighbors (KNN), and Light Gradient Boosting Machine (LightGBM). Model performance was evaluated using the area under the receiver operating characteristic curve (AUC), with higher values indicating better discriminative ability. AUC values were compared between training and testing sets to assess model generalizability. An AUC of 0.5 indicates no discriminative ability (equivalent to random guessing), while 1.0 indicates perfect discrimination.

### Model interpretability analysis

SHAP (SHapley Additive exPlanations) analysis was applied to the best-performing model to interpret feature contributions. SHAP values quantify each feature’s marginal contribution to individual predictions based on Shapley values from cooperative game theory (18). SHAP summary plots were generated to visualize feature importance rankings and the direction of feature effects. SHAP dependence plots were constructed to examine the relationship between individual feature values and their SHAP contributions.

### Software and packages

All analyses were performed using R 4.5 and Python 3.9 version software. Continuous variables were compared using the two-sample *t*-test, and categorical variables using the Chi-squared test. To reduce overfitting and remove redundant variables, the Least Absolute Shrinkage and Selection Operator (LASSO) regression was applied to variables with *p* < 0.01 in the univariate analysis. The optimal penalty parameter (*λ*) was determined via 10-fold cross-validation based on the 1-standard-error (1-se) rule. Machine learning models were implemented using scikit-learn 1.0, XGBoost 1.5, and LightGBM 3.3. SHAP analysis was conducted using the shap 0.40 package. A two-sided *p* < 0.05 was considered statistically significant.

## Results

### Baseline characteristics and univariate analysis of PASI100 response

A total of 11,134 patients with plaque psoriasis who received secukinumab treatment were included in the analysis. Among them, 4,593 patients (41.25%) achieved PASI100 response, while 6,541 patients (58.75%) did not. The baseline characteristics of the two groups are presented in Table 1. Among continuous variables, disease duration was significantly longer in non-response compared to response (*p* = 0.024). Additionally, BMI was significantly higher in non-response than response (*p* = 0.007). Notably, indices of baseline disease severity, including bBSA, bPASI, and bDLQI were consistently higher in non-response, with all comparisons yielding *p*-values < 0.001. Age did not differ significantly between the two groups (*p* = 0.059). For categorical variables, gender distribution revealed a small but statistically significant difference (*p* < 0.001), with 32% of response being female compared to 35% of non-response. The baseline disease severity as measured by bIGA differed markedly between groups (*p* < 0.001). Significant group differences were also found in socioeconomic variables; education background (*p* < 0.001) and job status (*p* < 0.001) differed significantly. Regarding clinical history, comorbidity status showed a modest difference (*p* < 0.001), while history of malignancy did not demonstrate significant difference (*p* = 0.2). Family history of psoriasis showed a strong association with PASI100 response (*p* < 0.001). Smoking habits were not significantly associated with PASI100 response (*p* = 0.089). In contrast, drug allergy history exhibited significant differences (*p* < 0.001), whereas allergic disease history was similar across groups (*p* > 0.9). The baseline disease situation (stable, worsening, relieving) was significantly different between groups (*p* < 0.001). Topical therapy acceptance rates were almost identical in both groups (*p* = 0.4). However, traditional systemic therapy was significantly less common among response (*p* = 0.003). Medical insurance status also revealed significant differences; those with insurance constituted 98% of non-response compared to 97% of response (*p* = 0.027). There were significant differences in disease status (*p* < 0.001), with 91% of non-response classified as recurrent compared to 87% of response. The biologic usage status was also notably different, with a higher proportion of biologic-experienced patients among non-response (*p* = 0.0335). Lastly, when considering geographic factors, the China region did not demonstrate significant differences in PASI100 response between non-response and response (*p* = 0.3).

**Table 1:** Baseline characteristics and univariate analysis of PASI100 response.

Variable	Non-response (*n =* 6,541)	Response (*n =* 4,593)	Statistic	*P*-value
Continuous variables, Mean ±SD				
Disease duration, years	12.89 ± 10.36	12.44 ± 10.36	t = 1.631	**0.024**
Age, years	45.39 ± 15.05	44.84 ± 15.12	t = 1.886	0.059
BMI, kg/m^2^	23.81 ± 3.04	23.65 ± 3.00	t = 2.715	**0.007**
bBSA, %	20.52 ± 15.81	15.11 ± 15.06	t = 18.137	**<0.001**
bPASI	13.17 ± 9.11	11.88 ± 9.47	t = 7.251	**<0.001**
bDLQI	11.93 ± 7.21	10.38 ± 6.91	t = 11.389	**<0.001**
Categorical variables, *N* (%)				
Gender			*χ*^2^ = 1143.888	**0.001**
Female	2,114 (32%)	1,618 (35%)		
Male	4,427 (68%)	2,975 (65%)		
bIGA			*χ*^2^ = 231.845	**<0.001**
Clear (0)	343 (5.2%)	406 (8.8%)		
Almost clear (1)	48 (0.7%)	63 (1.4%)		
Mild (2)	973 (15%)	841 (18%)		
Moderate (3)	3,032 (46%)	2,327 (51%)		
Severe (4)	2,145 (33%)	956 (21%)		
Education background			*χ*^2^ = 23.371	**<0.001**
High school	1,556 (24%)	1,265 (28%)		
Junior high school	2,491 (38%)	1,688 (37%)		
Bachelor	2,195 (34%)	1,414 (31%)		
Unknown	299 (4.6%)	226 (4.9%)		
Job			*χ*^2^ = 36.48	**<0.001**
Part-time	352 (5.4%)	341 (7.4%)		
Full-time (≥35 h)	4,362 (67%)	2,893 (63%)		
Unemployed	1,072 (16%)	722 (16%)		
Student	422 (6.5%)	347 (7.6%)		
Retired	333 (5.1%)	290 (6.3%)		
Comorbidity			*χ*^2^ = 7.509	**<0.001**
No	5,302 (81%)	3,780 (82%)		
Yes	579 (8.9%)	447 (9.7%)		
Unknown	660 (10%)	365 (7.9%)		
History of malignancy			*χ*^2^ = 1.392	0.2
No history	48 (0.7%)	44 (1.0%)		
History	6,493 (99%)	4,549 (99%)		
Family history			*χ*^2^ = 52.66	**<0.001**
No history	781 (12%)	706 (15%)		
History	4,944 (76%)	3,472 (76%)		
Unknown	816 (12%)	415 (9.0%)		
Smoking habits			*χ*^2^ = 6.508	0.089
Never	4,677 (72%)	3,226 (70%)		
Former	313 (4.8%)	242 (5.3%)		
Current	1,282 (20%)	962 (21%)		
Occasional	269 (4.1%)	163 (3.5%)		
Drug allergy history			*χ*^2^ = 37.191	**<0.001**
No allergy	5,624 (86%)	4,036 (88%)		
Allergy	202 (3.1%)	195 (4.2%)		
Unknown	715 (11%)	362 (7.9%)		
Allergic disease history			*χ*^2^ = 0.002	>0.9
No allergy	144 (2.2%)	101 (2.2%)		
Allergy	6,397 (98%)	4,492 (98%)		
Disease situation			*χ*^2^ = 36.098	**<0.001**
Stable	4,631 (71%)	3,009 (66%)		
Worsening	1,390 (21%)	1,199 (26%)		
Relieving	520 (7.9%)	385 (8.4%)		
Marital status			*χ*^2^ = 1.432	0.2
Unmarried	5,377 (82%)	3,734 (81%)		
Married	1,164 (18%)	859 (19%)		
Topical therapy			*χ*^2^ = 0.726	0.4
Not accepted	1,736 (27%)	1,185 (26%)		
Accepted	4,805 (73%)	3,408 (74%)		
Traditional systemic therapy			*χ*^2^ = 9.133	**0.003**
Not accepted	3,225 (49%)	2,399 (52%)		
Accepted	3,316 (51%)	2,194 (48%)		
Medical insurance			*χ*^2^ = 4.894	**0.027**
No insurance	136 (2.1%)	126 (2.7%)		
With insurance	6,405 (98%)	4,467 (97%)		
Disease status			*χ*^2^ = 38.375	**<0.001**
Recurrent	5,938 (91%)	4,005 (87%)		
Unknown	208 (3.2%)	180 (3.9%)		
New onset	395 (6.0%)	408 (8.9%)		
Biologic usage status			*χ*^2^ = 4.523	**0.0335**
Biologic-experienced	5,386 (82%)	3,847 (84%)		
Biologic-naïve	1,154 (18%)	746 (16%)		
China region			*χ*^2^ = 2.441	0.3
North area	1,806 (28%)	1,278 (28%)		
South area	4,219 (65%)	2,917 (64%)		
Unknown	516 (7.9%)	398 (8.7%)		

SD, standard deviation; BMI, body mass index; bBSA, baseline body surface area; bPASI, baseline Psoriasis Area and Severity Index; bDLQI, baseline Dermatology Life Quality Index; bIGA, baseline Investigator Global Assessment. Bold values indicate statistical significance (*p*<0.05).

Overall, variables reflecting baseline disease severity (bBSA, bPASI, bDLQI, bIGA), as well as gender, education background, job status, family history, drug allergy history, disease situation, traditional systemic therapy, medical insurance status, disease status and biologic usage status were demonstrated statistically significant differences between PASI100 response and non-response and were considered candidates for subsequent feature selection and model development (Table 1).

### Feature selection results

**Figure 1:**
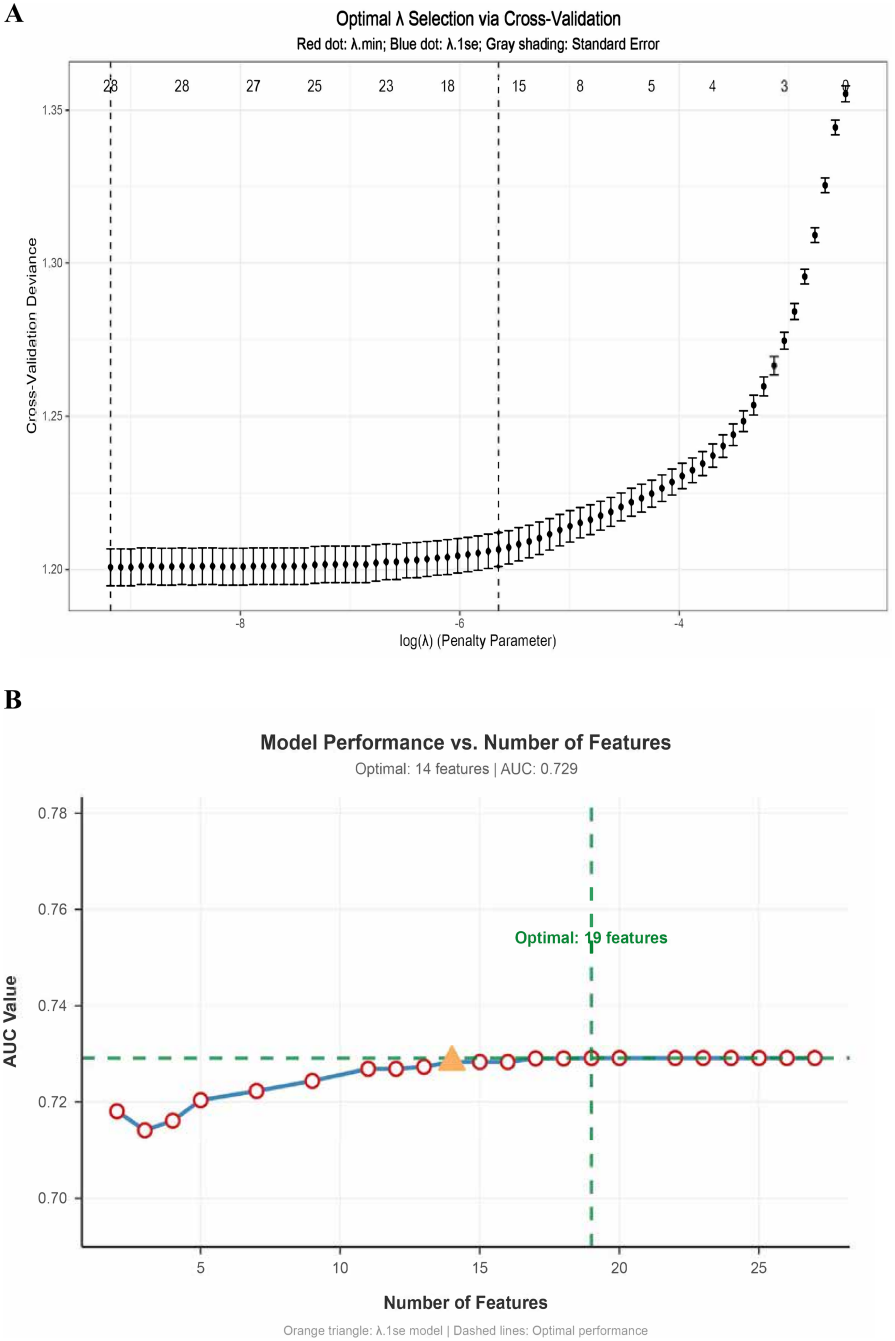
Feature selection using LASSO regression. **(A)** The binomial deviance (measured on the y-axis) versus the logarithmic transformation of the regularization parameter [log(Lambda), x-axis]. The red dots indicate the deviance for the optimal complexity, and error bars represent standard deviations. As Lambda increases, the binomial deviance stabilizes, suggesting an optimal model fit is reached at a specific value. **(B)** The relationship between the Area Under the Curve (AUC) value and the number of features used in the LASSO variable selection. The optimal AUC is achieved with 19 features, with an alternative model showing comparable performance at 14 features (identified by λ.1se). The green dashed line indicates the threshold for acceptable AUC performance.

Variables with *p* < 0.05 in univariate analysis were entered into LASSO regression for feature selection. Figure 1A shows the relationship of the binomial deviance (measured on the y-axis) versus the logarithmic transformation of the regularization parameter [log(Lambda), x-axis] and the number of features included in the model, and the optimal binomial deviance point indicated by 14 features. The maximum AUC was achieved with 30 features, while the *λ*.1se criterion selected 14 features (Figure 1B). Considering model performance, clinical interpretability, and practical applicability, 5 core predictive variables were ultimately retained: Gender, bIGA, bBSA, bPASI and bDLQI.

### Machine learning model performance comparison

**Figure 2:**
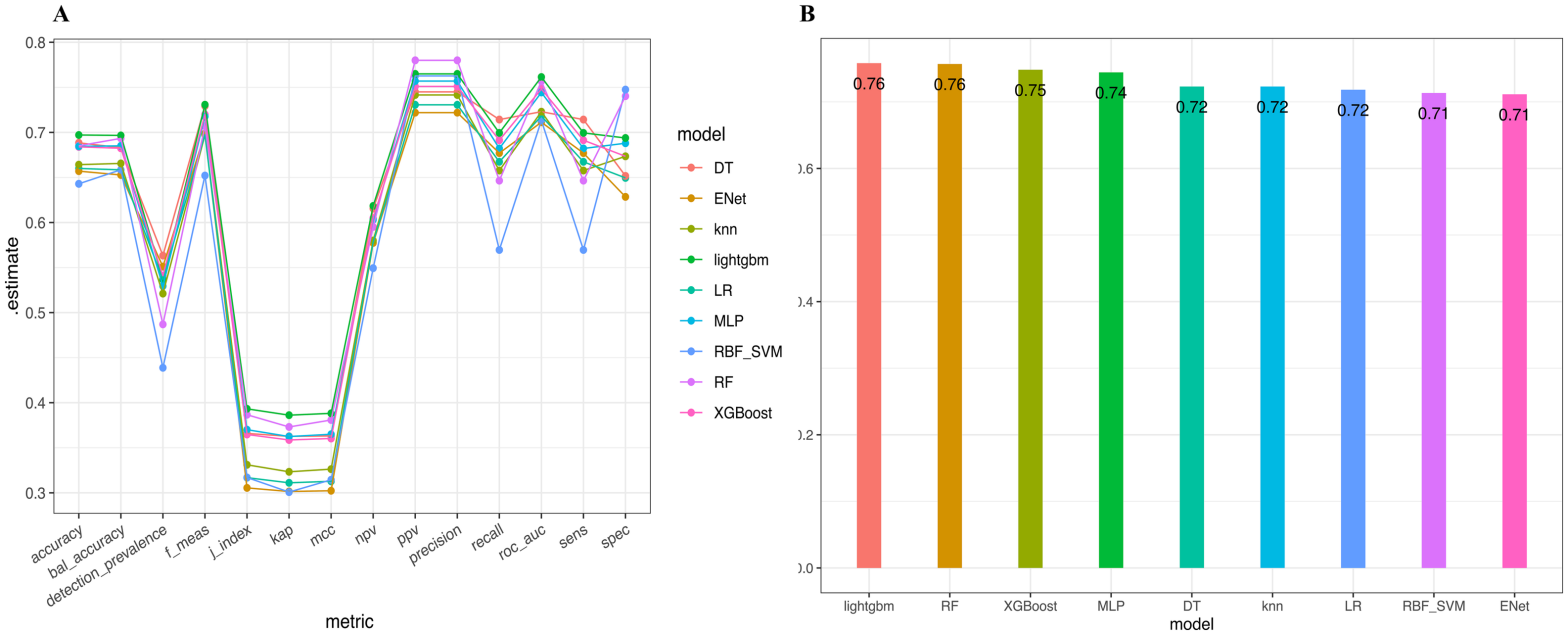
Performance comparison of machine learning models. **(A)** The performance estimate of various machine learning models across multiple evaluation metrics. The y-axis represented the estimate values, while the x-axis listed different metrics used for evaluation, including accuracy, balanced accuracy, detection prevalence, F-measure, the L-index, kappa (kap), Matthew’s correlation coefficient (mcc), negative predictive value (npv), positive predictive value (ppv), precision, recall, ROC AUC (roc_auc), sensitivity (sens), and specificity (spec). Models were color-coded: ENet - Elastic Net (Orange), knn - K-Nearest Neighbors (Yellow), lightgbm - Light Gradient Boosting Machine (Green), LR - Logistic Regression (Blue), MLP - Multi-Layer Perceptron (Cyan), RBF_SVM - RBF Support Vector Machine (Dark Blue), RF - Random Forest (Violet), XGBoost - XGBoost (Magenta). **(B)** Receiver Operating Characteristic (ROC) Area Under the Curve (AUC) comparison of machine learning models on the testing data. The y-axis showed the ROC AUC values, while the x-axis indicated the workflow rank for each model. Each point represented the mean ROC AUC with associated standard deviation error bars. Models were color-coded similarly to **(A)**, enhancing visual comparison of performance across different metrics.

**Figure 3:**
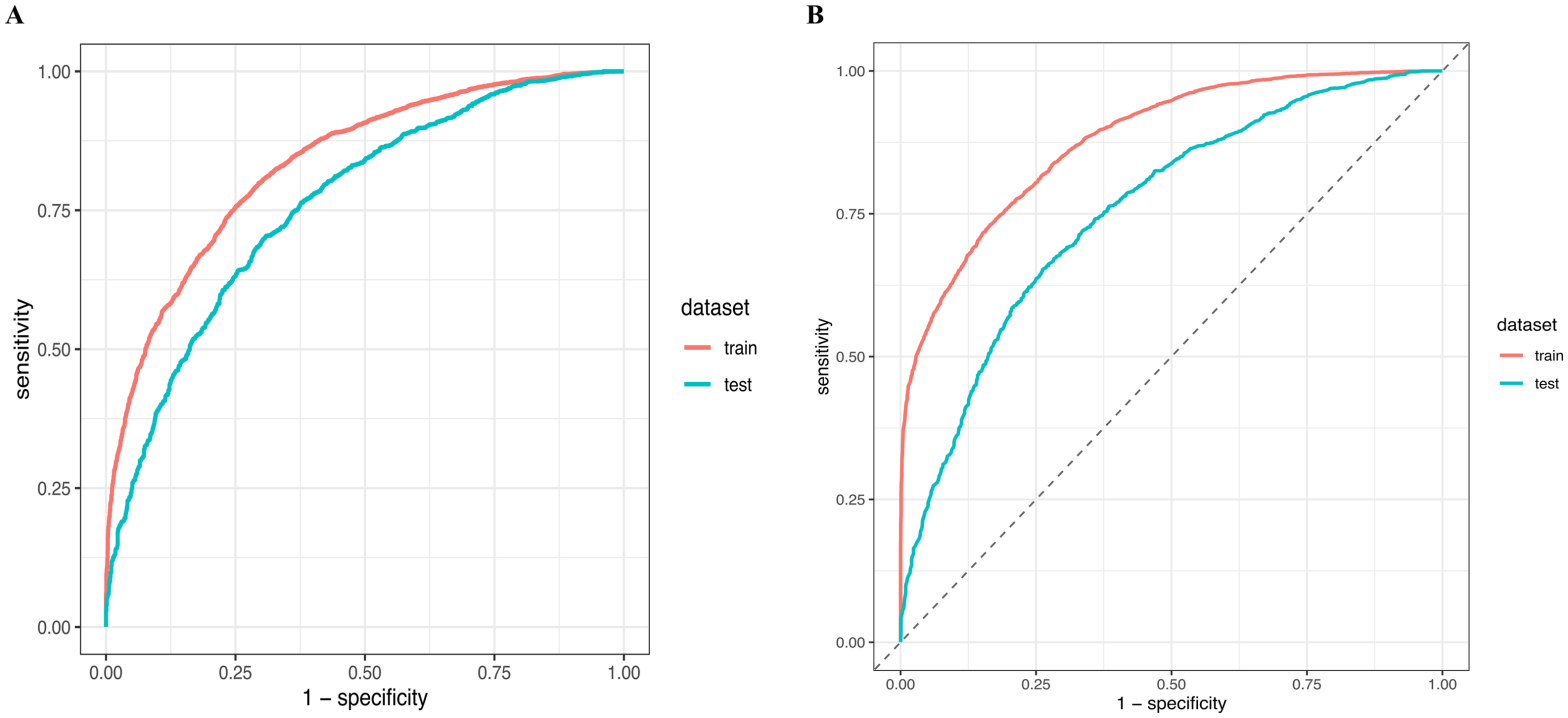
ROC curve analysis for model performance. **(A,B)** Receiver Operating Characteristic (ROC) curves for the training (red line) and test (blue line) datasets demonstrating the model’s sensitivity versus 1-specificity at various threshold settings. **(A)** Light Gradient Boosting Machine; **(B)** Random Forest.

The performance estimates of several machine learning models across different evaluation metrics were used, including accuracy, balanced accuracy, detection prevalence, F-measure, L-index, kappa (kap), Matthew’s correlation coefficient (mcc), negative predictive value (npv), positive predictive value (ppv), precision, recall, ROC AUC (roc_auc), sensitivity (sens), and specificity (spec). The results indicate that the performance of different models is relatively consistent, with most models exhibiting estimate values around the 0.7 mark across key metrics. Notably, the Random Forest and LightGBM models demonstrated particularly robust performance, achieving among the highest estimates across various metrics (Figure 2A). A summary of the average performance across models were showed in Figure 2B, showcasing average estimate values for each model. The bar chart demonstrates that both Random Forest and XGBoost scored were 0.76, respectively, indicating their superior predictive capabilities. Other models, including MLP, DT, and RBF-SVM, achieved average scores close to 0.72, while Elastic Net and K-Nearest Neighbors rounded out the performance with estimates of approximately 0.71 (Table 2; Figure 3).

**Table 2:** Performance comparison of machine learning models for predicting PASI100 response.

Model	Training AUC	Testing AUC
Light Gradient Boosting Machine	**0.834**	**0.761**
Random Forest	**0.879**	**0.757**
eXtreme Gradient Boosting	0.819	0.748
Multi-layer Perceptron	0.764	0.744
Decision Tree	0.731	0.723
K-Nearest Neighbors	0.813	0.723
Logistic Regression	0.721	0.72
Radial Basis Function Support Vector Machine	0.715	0.713
Elastic Net	0.715	0.711

AUC, area under the receiver operating characteristic curve. Bold text indicates the best machine learning model.

### SHAP interpretability analysis of Random Forest model

**Figure 4:**
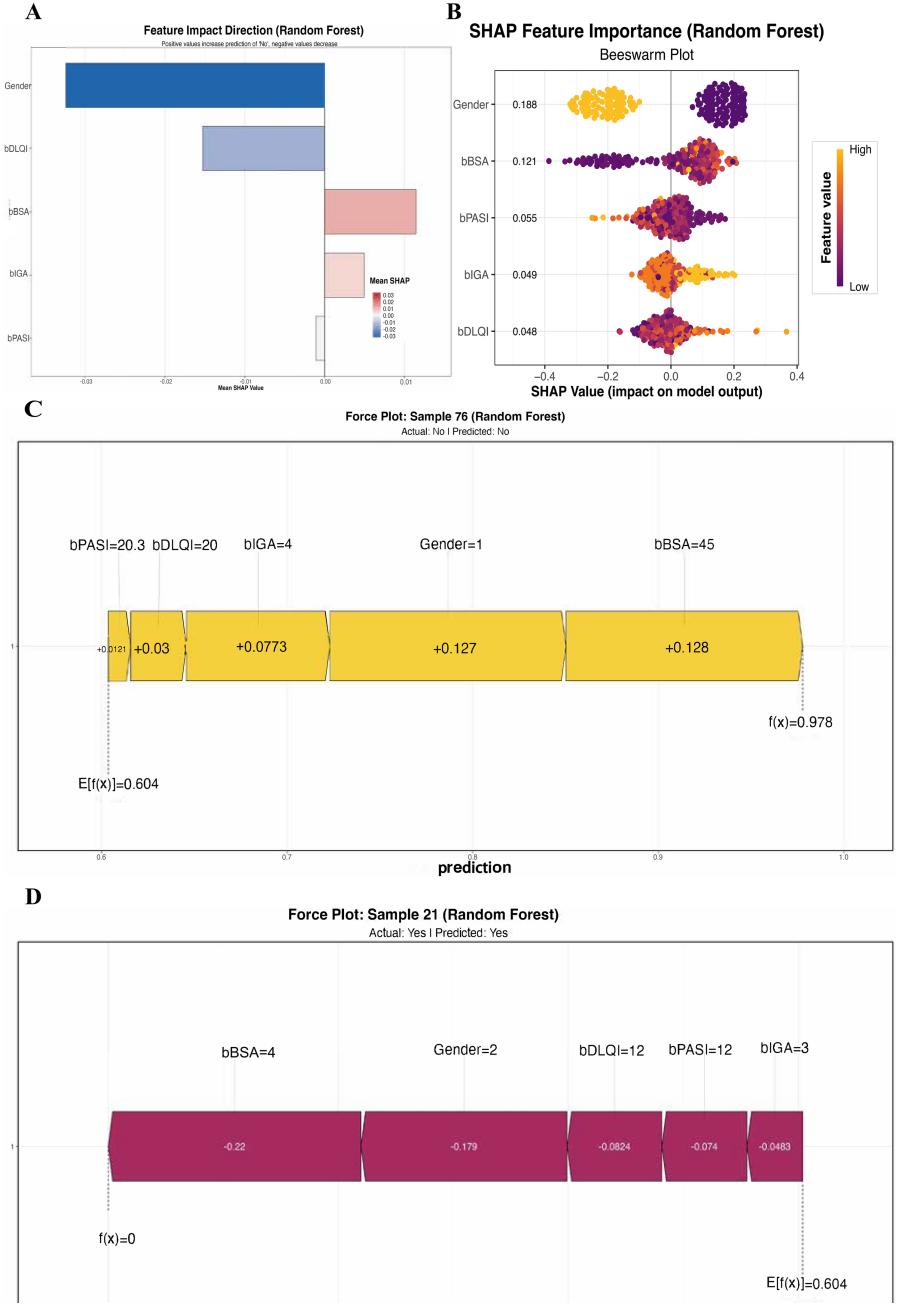
SHAP analysis of feature importance in Random Forest model. **(A)** Feature Impact Direction: Bar graph depicting the mean SHAP values for various features affecting PASI100 response predictions. Positive values (indicated in blue) suggest an increase in the likelihood of achieving PASI100, while negative values (indicated in pink) suggest a decrease. **(B)** SHAP Feature Importance: Beeswarm plot representing the SHAP values for individual features across multiple samples. Each point reflects the SHAP value for each feature, color-coded by the feature's value (with orange indicating higher values and purple indicating lower values). **(C)** Individual Prediction Analysis for Sample 76: Force plot illustrating the contribution of each feature to the model's prediction for Sample 76. **(D)** Individual Prediction Analysis for Sample 21.

SHAP analysis was applied to the Random Forest model to interpret feature contributions. Figure 4A illustrated the feature impact direction derived from a Random Forest model, presented as a bar graph that depicted the mean SHAP values. This graph highlighted the average contribution of each feature to the model’s predictions. Notably, Gender and bBSA emerged as the most influential features, with positive contributions indicating that increases in these values correlated with a higher probability of achieving a PASI100 response. In contrast, features such as bPASI, bIGS, and bDLQI demonstrated negative contributions, suggesting that higher values in these metrics decreased the likelihood of achieving PASI100. Figure 4B presented a beeswarm plot that showcased the SHAP feature importance for the Random Forest model. Each point in the plot represented the SHAP value for an individual feature across various samples and was color-coded to reflect the feature value. Importantly, Gender and bBSA exhibited substantial positive SHAP values of 0.188 and 0.121, respectively, signifying their significant impact on the model’s predictions and their positive influence on the likelihood of achieving PASI100. Conversely, features like bPASI, bIGA, and bDLQI displayed lower SHAP values, indicating a relatively minor but still negative effect on the prediction. Figure 4C displayed a force plot for Sample 76, outlining the model’s prediction process. The actual outcome for this sample was classified as “No” for achieving PASI100, which aligned with the model’s prediction of the same. The SHAP values illustrated how each feature contributed to this prediction: bBSA (45) provided a positive contribution (+0.128), while Gender (1, representing female) also contributed positively (+0.127). Other features, such as bPASI (20.3) and bDLQI (20), made minor positive contributions, ultimately leading to a final prediction score of f(x) = 0.978. In Figure 4D, the force plot for Sample 21 revealed a different scenario, as this sample was predicted to achieve PASI100 (“Yes”), coinciding with the actual outcome. Here, bBSA (4) exhibited a significant negative contribution (−0.22), while Gender (2, representing male) also had a negative impact (−0.179). Other features, including bDLQI (12), bPASI (12), and bIGA (3), similarly provided negative contributions, which together resulted in a final prediction score of f(x) = 0.

### SHAP interpretability analysis of light gradient boosting machine model

**Figure 5:**
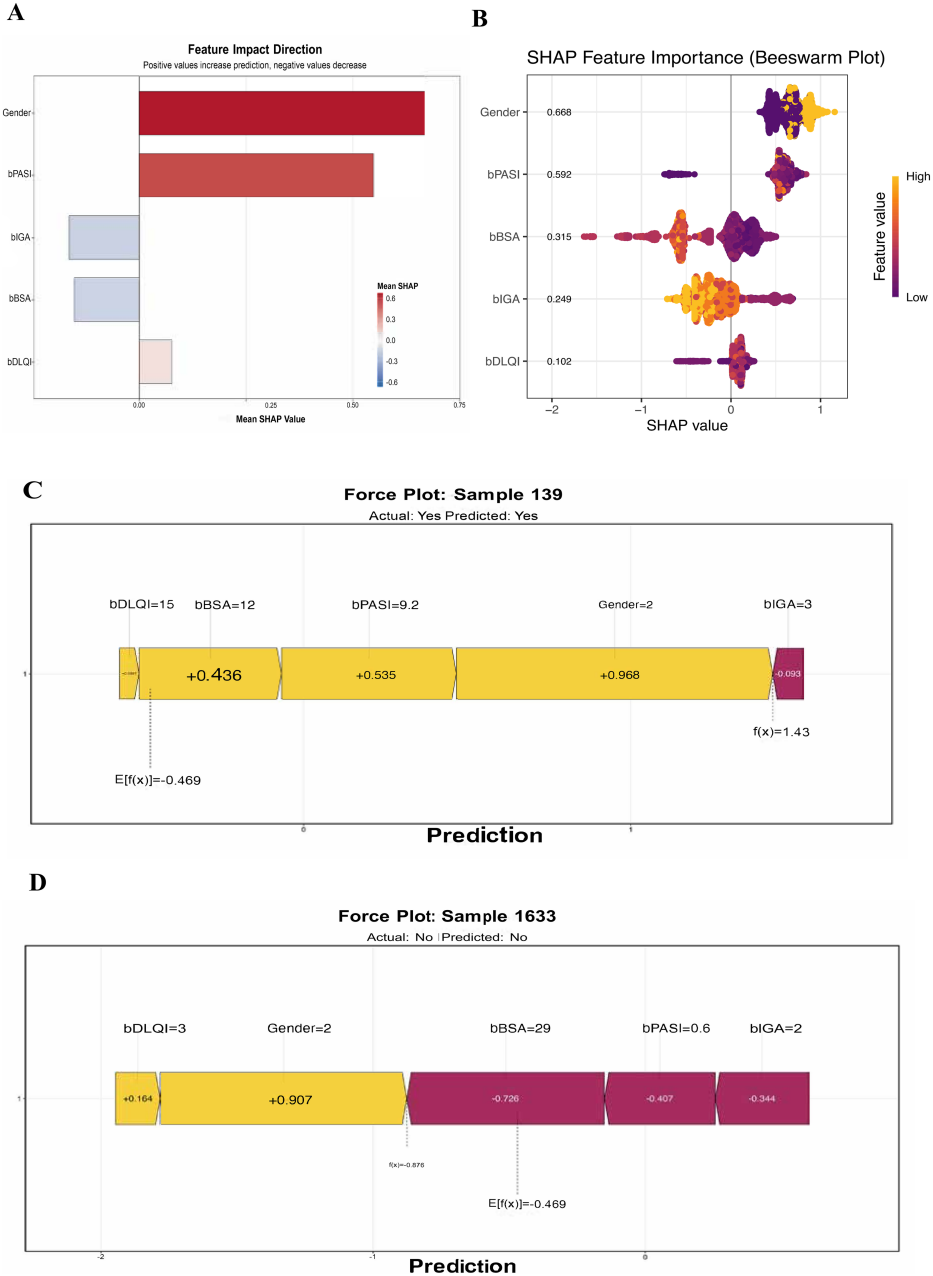
SHAP analysis of feature importance in light gradient boosting machine model. **(A)** Feature impact direction: bar graph depicting the mean SHAP values for various features affecting PASI100 response predictions. Positive values (indicated in blue) suggest an increase in the likelihood of achieving PASI100, while negative values (indicated in pink) suggest a decrease. **(B)** SHAP Feature Importance: Beeswarm plot representing the SHAP values for individual features across multiple samples. Each point reflects the SHAP value for each feature, color-coded by the feature's value (with orange indicating higher values and purple indicating lower values). **(C)** Individual prediction analysis for sample 139: Force plot illustrating the contribution of each feature to the model's prediction for Sample 139. **(D)** Individual prediction analysis for sample 1633.

SHAP analysis was applied to the Light Gradient Boosting Machine model to interpret feature contributions. Notably, Gender had the highest mean SHAP value, indicating it was the most influential factor in increasing the prediction likelihood. Other influential features included bPASI and bIGA, both of which also contributed positively. In contrast, features such as bBSA and bDLQI were less influential, with bDLQI showing a negative mean SHAP value, suggesting it negatively impacted the prediction (Figure 5A). As illustrated in Figure 5B, gender demonstrated the highest SHAP value among the features, with a mean SHAP score of 0.666, signaling a strong positive influence on predictions. bPASI and bBSA followed, with SHAP values of 0.592 and 0.315, respectively. bIGA had a moderate positive impact (0.249), while bDLQI exhibited a negative value of −0.102, indicating that an increase in bDLQI was associated with a lower likelihood of the predicted positive outcome. The sample 139 was displayed as Figure 5C, demonstrating the model’s prediction mechanics. The actual outcome for this sample was categorized as “Yes,” and the model also predicted “Yes.” The contributions of various features to the prediction were highlighted. Positive contributions were shown in yellow, while the overall prediction score was indicated on the right side of the plot. Specifically, bPASI contributed +0.535, Gender contributed +0.968, bBSA contributed +0.436, and bDLQI contributed +0.436. The expected base value, denoted as E[f(x)], was −0.469, summing the contributions to yield a final prediction score of f(x) = 1.43, confirming a positive prediction. The other Sample 1,633 was presented as Figure 5D, illustrating a different prediction trajectory. The actual outcome for this sample was “No,” and the model similarly predicted “No.” In this case, the contributions were visualized, with negative contributions shown in purple. Here, bBSA had a significant negative impact (−0.728), while bPASI (−0.407), bIGA (−0.344), and Gender (−0.907) all contributed negatively. The expected base value E[f(x)] was also −0.469, and the final prediction score was f(x) = −0.876, indicating a strong prediction that aligned with the actual outcome.

## Discussion

The finding that baseline disease severity indicators (bPASI, bBSA, bIGA, and bDLQI) were the most important predictors of PASI100 response aligns with previous research. In a German registry study of over 3,000 patients, Augustin et al. (19) reported that lower baseline PASI was associated with higher rates of PASI90 and PASI100 achievement with biologic therapy. Reich et al. (20, 21) confirmed in a meta-analysis that baseline disease severity was a significant predictor of IL-17 inhibitor efficacy. Several pathophysiological mechanisms may explain the inverse relationship between baseline severity and PASI100 response. First, patients with severe psoriasis have higher levels of IL-17A expression and more complex inflammatory cascades in lesional skin; blocking IL-17A alone may be insufficient to completely reverse established pathological changes (22). Second, severe psoriasis is frequently associated with metabolic syndrome and obesity, which may affect drug pharmacokinetics and produce additional pro-inflammatory signals that attenuate treatment effects (23). Third, more extensive tissue remodeling in severe lesions may require longer duration for complete epidermal restoration, even after inflammation is controlled. A noteworthy aspect of our findings is the identification of gender as a leading predictor of PASI100 response, which emerged prominently in both SHAP analyses. This observation may reflect underlying biological differences in immune response and disease manifestations based on sex (24, 25). However, it is crucial to recognize the potential for confounding factors that may contribute to this result. Disease characteristics—such as severity and duration of psoriasis—are known to differ between genders (26, 27), potentially influencing treatment efficacy. Furthermore, historical treatment biases may exist, with male and female patients encountering differences in access to specific therapies and overall treatment approaches (28, 29). Socioeconomic variables, including income, education, and healthcare access, may also impact treatment adherence and outcomes differently across genders (30, 31). Expanding our discussion to include these variables provides a more nuanced understanding of how gender influences treatment response in psoriasis.

Among the eight algorithms compared, Random Forest and Light Gradient Boosting Machine outperformed traditional Logistic Regression, supporting the value of machine learning in clinical prediction modeling. These algorithms can capture complex non-linear interactions between variables, potentially improving predictive accuracy (32). However, Random Forest exhibited little overfitting tendency, with a 0.122 gap between training and testing AUC. Future studies should explore strategies to mitigate overfitting, such as enhanced regularization, stricter cross-validation protocols, or larger validation datasets. In contrast, Light Gradient Boosting Machine showed perfect generalization stability despite lower discriminative ability, which may be preferable in clinical settings requiring model robustness. The integration of SHAP analysis addressed the interpretability challenge of machine learning models. Through SHAP summary and dependence plots, clinicians can intuitively understand each factor’s contribution to individual predictions, which is essential for clinical acceptance and practical implementation (33).

This study has several limitations. First, external validation in independent cohorts was not conducted, which necessitates further confirmation of the model’s generalizability across different populations. Additionally, only baseline static variables were included in the analysis; dynamic factors during treatment, such as early response trajectories and drug concentration, were not considered, despite their potential importance in predictive modeling. Although the testing AUC of 0.761 exceeds random prediction, it indicates that there is still room for improvement, potentially through the inclusion of additional predictors such as genetic markers or biomarkers. Future research should consider the following: (1) Incorporating genetic variables (e.g., HLA typing, IL-17A polymorphisms) and biomarkers (e.g., serum IL-17A levels, C-reactive protein) to enhance predictive performance; (2) Developing dynamic prediction models that integrate early treatment response data (e.g., responses at week 4 or week 12) to better predict final PASI 100 achievement; (3) Conducting multicenter prospective validation studies to assess the model’s applicability across diverse populations; (4) Creating user-friendly clinical decision support tools or mobile applications to facilitate the translation of research findings into clinical practice; (5) Refining efficacy analyses for disease subgroups, following study approaches similar to the efficacy of Guselkumab (34), which was consistent across various subpopulations, both on the skin and systemically and (6) Utilizing DLQI 100 as an additional treatment goal in the predictive model alongside PASI 100, providing a more comprehensive measure of treatment success.

## Conclusion

This study successfully developed a Random Forest and Light Gradient Boosting Machine-based prediction model for PASI100 response to secukinumab in a large real-world cohort of 11,134 psoriasis patients. Five core predictive factors were identified through LASSO regression, with baseline disease severity indicators (bPASI, bBSA, bIGA, bDLQI) and gender demonstrating the greatest predictive importance. These findings provide evidence-based support for personalized treatment decision-making and patient selection strategies in psoriasis management.

## Data Availability

The original contributions presented in the study are included in the article/supplementary material, further inquiries can be directed to the corresponding authors.
